# The business of quality: analysing the association between healthcare quality improvements and patient utilization and staff numbers in sub-Saharan Africa

**DOI:** 10.3389/frhs.2026.1856921

**Published:** 2026-08-04

**Authors:** Gloria P. Gómez-Pérez, Daniella Brals, Aafke E. de Graaff, Albena Sotirova, Ibironke Dada, Bonifacia Benefo Agyei, Peter Risha, Elizabeth Bonareri, John T. Dekker, Tobias F. Rinke de Wit, Nicole Spieker, Wendy Janssens

**Affiliations:** 1 PharmAccess Foundation, Amsterdam, Netherlands; 2 Amsterdam Institute for Global Health and Development, Amsterdam, Netherlands; 3 Department of Global Health, Amsterdam University Medical Centers, Location University of Amsterdam, Amsterdam, Netherlands; 4 PharmAccess Nigeria, Lagos, Nigeria; 5 PharmAccess Ghana, Accra, Ghana; 6 PharmAccess Tanzania, Dar es Salaam, Tanzania; 7 PharmAccess Kenya, Nairobi, Kenya; 8 Faculty of Economics, Vrije Universiteit, Amsterdam, Netherlands

**Keywords:** business performance, healthcare facilities, quality of care, sub-Saharan Africa, universal health converge

## Abstract

**Background:**

An estimated 5 to 8 million people die each year in low- and middle-income countries due to poor-quality care. Although quality improvements in healthcare facilities are feasible, costs are often mentioned as a prohibiting factor. However, if quality improvements increase patient trust and demand, this might translate into increased visits and higher revenues for providers, enabling further investments in quality. This study assesses the potential business case of quality improvement in sub-Saharan Africa.

**Methods:**

We analysed quality assessment scores and business performance indicators of 483 public and private facilities in Tanzania, Kenya, Ghana, and Nigeria. Using longitudinal data collected at least 18 months apart, we examined associations between changes in quality scores and changes in patient visits and staff levels, as proxies for business performance, based on linear regression analyses of baseline and follow-up data.

**Results:**

Quality improvements were significantly associated with increased patient visits [*β*=10.1, 95% confidence interval (CI): 3.6‒16.6]. Facilities with higher baseline quality scores experienced larger increases in patient visits, as did those who started with lower baseline patient volumes. Longer follow-up periods were also associated with larger increases. Similarly, quality improvements were associated with increases in staff numbers (*β*=0.25, 95% CI: 0.13‒0.38); again, facilities with higher quality scores and lower staff numbers at baseline experienced larger increases.

**Conclusions:**

Quality improvements are positively associated with our business performance proxies, patient visits and staff numbers, suggesting a potential business case for QI in these facilities, although causality cannot be established. Findings suggest that time is needed for this association to materialize. Targeted initial investments might be needed to support lower-performing facilities in realizing business performance gains similar to those of facilities with higher quality scores, particularly where resources or capacity are limited. Public-private partnerships, along with innovative loan schemes, could help bridge these gaps.

## Background

Access to healthcare in low- and middle-income countries (LMICs) has improved considerably during the last decades, making poor-quality care a bigger barrier to reducing mortality than insufficient access. It has been estimated that 60% of deaths from conditions amenable to healthcare are due to poor quality, with 8 million people in LMICs dying per year from conditions that should be treatable by a basic healthcare system. Apart from affecting patient outcomes, poor quality care leads to unnecessary social and economic losses, lack of patient trust, waste of resources, and catastrophic expenditures (1). Increasing access to care will be ineffective in improving health outcomes and universal health coverage (UHC) as long as quality of care is not simultaneously addressed. However, improving quality of care is difficult in LMICs due to limited funding and human resources, low patient trust and empowerment, faltered infrastructure, and poor regulation when substandard care is delivered. Moreover, nearly 40% of health care facilities in LMICs lack access to clean water, and nearly 20% lack sanitation (2), further reducing healthcare quality.

These challenges are aggravated in sub-Saharan Africa (SSA) which has 14% of the world's population (3), but carries the highest disease burden worldwide measured in disability-adjusted life-years (DALYs) (4). With less than 1% of global health expenditures, only 3% of the world's health workers (5), and difficulties to ensure the level of infrastructure required to provide good quality care, SSA cannot provide even the most basic health care to a large proportion of its people, let alone ensure the level of quality needed for better health outcomes. Per capita public expenditures on health in some countries of SSA, measured by purchasing power parity (PPP), were below USD 40 in 2018 (6), compared to a World Health Organization (WHO)-recommended minimum level of USD 86, and a level of at least USD 200 per capita investment needed to achieve significant improvements in financial protection of the population against expenditures on health (7). In addition, the WHO estimates that quality of care in SSA is at only 63% of what is feasible given its health expenditures, with marked variations between countries from 25% to 94% (8).

Although an estimated 50% of healthcare in Africa is provided by the private sector, reaching both high- and low-income groups, with figures up to 77% in some SSA countries (9), official development assistance has bypassed the private healthcare sector for many years. The WHO has recognized the private sector as a key partner in achieving UHC in Africa (10). The need to involve the private sector in health care provision has been further underscored during the COVID−19 pandemic (11, 12).

To improve quality of care, both financial resources and capacity building are required. Financial institutions can help with initial investments (e.g., through digital loans) (13), but financial sustainability requires facilities to keep their business afloat by securing sufficient and continuous patient numbers to ensure sustained revenue streams. Patient numbers in turn crucially depend on the quality of care provided to keep patients’ experience positive. If patients do not sufficiently value the provided services at the requested prices, utilization will be low. In the African region, the WHO reports that a wide-spread perception of poor experiences with the healthcare process affects the demand for services (8). In addition, low quality of care hampers the empanelment of clinics in insurance schemes, further undermining facilities' ability to secure a substantial number of patients. These processes complete and perpetuate a vicious circle of low demand and poor supply (14, 15).

Evidence from healthcare facilities in high-income countries supports the business case for quality improvement (QI), showing that investing in QI work can create financial revenues for healthcare organizations over time, have beneficial organizational effects, and improve outcomes for patients (16). The latter requires enhanced financial and economic management, and the delivery of care across all the dimensions of quality (17). Similarly, several case studies of tertiary public and private hospitals in Nigeria and Kenya show how they have improved their business performance as a result of improvements in quality, for instance translating into reduction of costs associated to legal claims, or increased patient satisfaction and demand after automated implementation of clinical guidelines (18, 19). Over the years, these facilities had increased their number of patients visits, with growing numbers of staff and services offered, and improved healthcare outcomes (18). These hospitals had in common that they are large facilities with a strong financial and organization system, which allowed them to develop an economically sustainable QI model. After reaching certain quality level, these hospitals were empanelled in insurance schemes that provided a secure stream of money to self-finance continuous QI investments.

Beyond these and other few documented cases, very limited evidence exists from SSA on whether improved quality of care in public and private clinics is indeed associated with improved business performance. Such evidence would strengthen the business case of QI in Africa. To our knowledge, this paper is the first to investigate the association between improvements in quality and in business performance indicators for public and private small- and medium- healthcare providers in Africa, even if we cannot establish causality with the available data. We analyse changes in quality assessments for 483 public and private healthcare facilities in four countries in SSA and relate those to changes in patient numbers and numbers of health care staff, used as proxies for business performance.

## Methods

### Safecare methodology

Our study focuses on clinics that participated in the SafeCare QI program. The latter encompasses a QI and stepwise certification approach developed in 2009 by PharmAccess Foundation, the Joint Commission International (JCI), and the Council for Health Service Accreditation of Southern Africa (COHSASA) to provide innovative health care standards, and a QI process broken into achievable, measurable steps to facilitate incremental improvement of quality (20). SafeCare standards are accredited by the International Society for Quality in Health Care External Evaluation Association, established by the International Society for Quality in Health Care in 2018 as an independent entity to provide its external evaluation services, and also abbreviated as ISQua-EEA. SafeCare standards allow the evaluation and rating of healthcare quality in small- and medium-size facilities in LMICs. Its quality assessment is organized along 13 service elements that constitute the various medical and non-medical aspects of health care delivery, comprising a total of 753 SafeCare criteria and summarized into an overall score (20). After a baseline assessment, a tailored quality improvement plan (QIP) is developed for each facility to guide the process of improvement. The QIP is built upon the identification of priorities for each healthcare facility. SafeCare also provides technical assistance to help achieve the provided QIP. This is tailored to the context of each healthcare centre based on its financial and human resource capacities, and the baseline quality level. Finally, based on the SafeCare scores, each facility receives a rating ranging from level 1 (very modest quality) to level 5 (continuous QI systems in place). The rating allows benchmarking between facilities and helps providers to track their progress. It also provides policymakers, patients, and donors in the healthcare system with data for informed decision-making and resource allocation.

In addition to the SafeCare program, PharmAccess Foundation offers healthcare facilities access to small- and medium-sized loans through the Medical Credit Fund (MCF) (21) to finance QI implementations, as well as business support training.

### Study design and sampling

**Figure 1:**
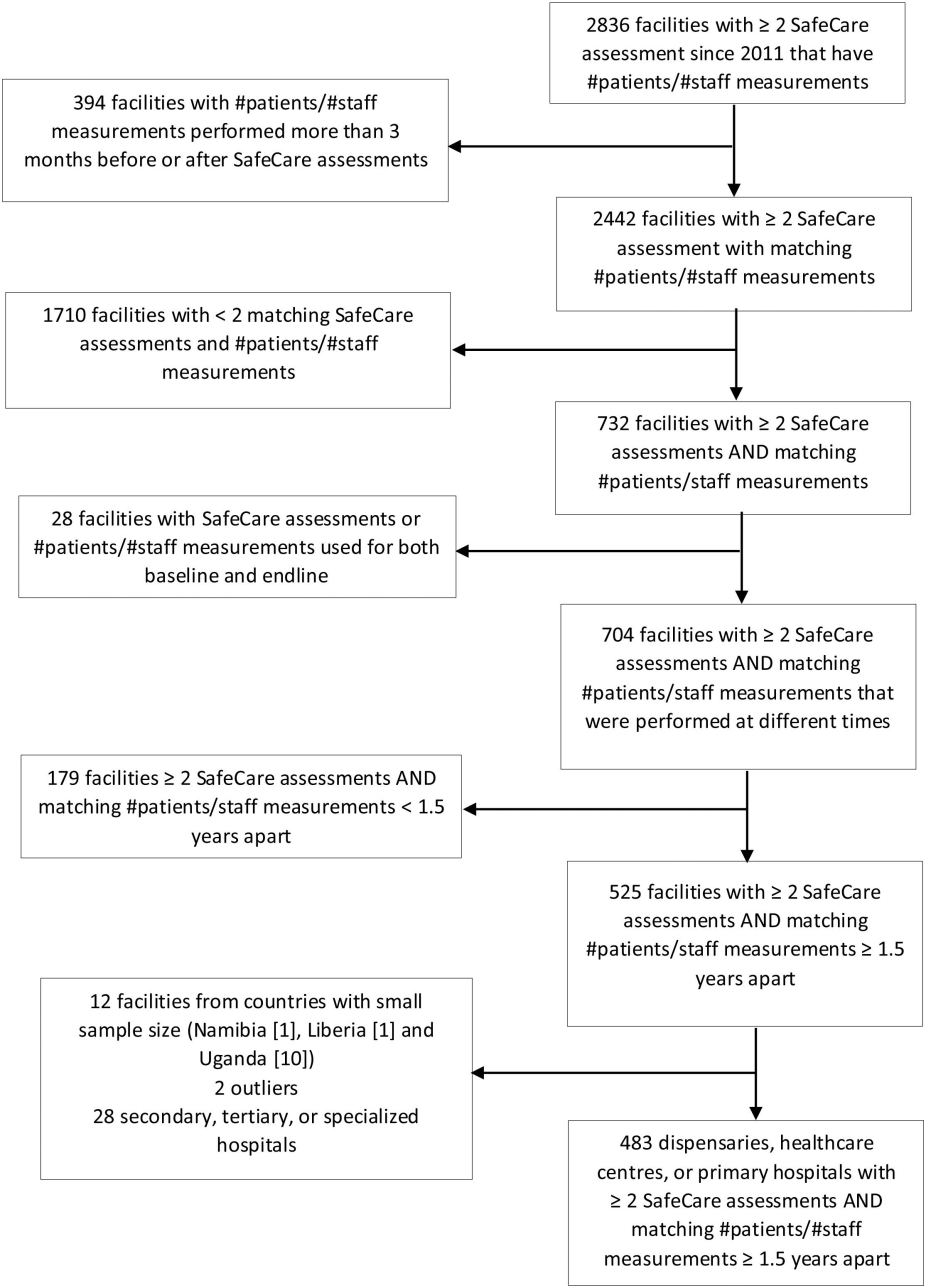
Study flowchart.

We conducted a retrospective cohort study to investigate the association between the changes in quality of care measured by SafeCare standards and changes in the business performance of healthcare facilities measured by the number of patient visits and number of medical staff. The potential study population consisted of 2,836 health care facilities from Tanzania, Ghana, Kenya, Nigeria, Namibia, Liberia, and Uganda participating in the SafeCare program since 2011 and with at least two SafeCare assessments. Facilities were only included in the analysis if they also had ≥ 2 measurements of patient visits and number of medical staff that were performed no more than 3 months before or after the baseline quality assessment, and no more than 3 months before or 6 months after the follow-up quality assessment. In addition, the baseline and follow-up quality assessments had to be ≥18 months apart for facilities to be included. Moreover, only dispensaries, health care centres and primary hospitals were included. Secondary, tertiary, and specialized hospitals in the dataset generally had very high baseline SafeCare scores, such that the magnitude of further potential score improvement was very small for these facilities. Finally, facilities located in Namibia, Liberia, and Uganda were excluded because of the small number of centres per country full filling the inclusion criteria. A total of 483 facilities complied with the above inclusion criteria and were hence included in the analysis (Figure 1).

### Measurement of variables

Conventional business performance indicators include revenue growth, profitability, and operating margins, among others. However, obtaining this information from healthcare small and medium enterprises (SMEs) in low-resource settings is difficult, due to a lack of reliable administrative records and a reluctance to share financial data with third parties. Nevertheless, understanding the business performance of these facilities is important to assess the cost-effectiveness of QI interventions. As proxies for business performance, we therefore used the number of patient visits and the number of medical staff. The number of patient visits was calculated as the average of monthly patients visits reported over a six month-period prior to the facility survey data collection, and the number of medical staff was calculated as the average number of staff reported monthly over that same period (20). Staff included nurses and medical officers with a part-time or full-time employment contract in the facility at the time of the measurement. Changes in each outcome were calculated as the difference between the first and the last measurement per facility (in cases with more than two measurements). Patient volume is arguably the stronger of the two proxies, as it is generally linked to patient satisfaction, which has in turn been associated with hospital revenue and lower costs (22, 23). Staff numbers, in turn, are commonly used alongside turnover as a standard indicator of firm growth in the SME and economic development literature (24, 25), on the assumption that facilities expand their workforce as they grow. We acknowledge, however, that this assumption does not always hold: employment growth and turnover (or revenue) growth do not necessarily move in tandem, and staff growth can also reflect organizational inefficiency or rising operating costs rather than genuine business growth. We discuss this limitation further below.

SafeCare scores are based on the SafeCare assessments. Scores represent a punctuation of all 753 applicable criteria as fully compliant (2 points), partially compliant (1 point), or non-compliant (0.25 points) within each of 13 service elements. Scores are transformed to range from 1 to 100. More details have been published elsewhere (20). The quartiles of the baseline SafeCare score distribution in our sample were calculated to create four equally-sized quality score categories as follows: low [21–36], lower-medium [36–43], higher-medium, [43–54], and high [54–94]. QI was measured as the change in SafeCare score between the baseline and the last follow-up measurement. As an alternative quality measure, we also calculated scores based only on 38 out of the 753 SafeCare criteria in the category of business performance.

### Characteristics of facilities

483 facilities located in Tanzania, Kenya, Nigeria, and Ghana were included in this analysis as these facilities had at least two performance measurements that were 18 months apart with matching SafeCare scores as per inclusion criteria and were below the level of secondary hospital at baseline (Table 1). Forty-three percent of the facilities were dispensaries (small clinics that provide basic primary care consultations, that include diagnostic services and treatment for routine conditions), 39% were (primary) healthcare centres and 18% primary hospitals. The majority were private (58%), 34% faith-based and 8% public. Thirty seven percent were located in rural areas. Most facilities were from Tanzania (53%), 35% from Kenya, 9% from Nigeria and 3% from Ghana. Table 1 compares the characteristics of excluded and included facilities. The excluded facilities had lower medians of baseline numbers of patients visits and staff. The median SafeCare score was also lower in the excluded group, although the absolute difference was small. The proportion of dispensaries and hospitals was higher among the included facilities, as well as the proportions of private, faith-based, rural, and Tanzanian facilities. These differences should be taken into consideration when interpreting the results.

**Table 1:** Facilities characteristics at baseline.

Variables	Excluded^a^	Included	*P-*value^a^
	(*N* = 2,353)	(*N* = 483)	
# Patient visits, median (IQR)	491 (841)	600 (1,000)	**<0**.**001**
# Staff, median (IQR)	13 (22)	15 (28)	**0**.**002**
Quality score, median (IQR)	42 (16)	43 (18)	**<0**.**001**
Quality score categories,^c^ *n* (%)			
Low [excluded (14–34); included (21–36)]	607 (25.8)	121 (25.1)	0.73
Lower medium [excluded (34–42); included (36–43)]	586 (24.9)	121 (25.1)	0.95
Higher medium [excluded (42–50); included (43–54)]	592 (25.2)	121 (25.1)	0.96
High [excluded (50–92); included (54–94)]	568 (24.1)	120 (24.8)	0.74
Facility level, *n* (%)			
Dispensary	827 (35.2)	207 (42.9)	**0**.**001**
Healthcare center	982 (41.8)	188 (38.9)	0.243
Primary hospital	288 (12.3)	88 (18.2)	**<0.001**
Secondary hospital	253 (10.8)	0 (0)	**<0.001**
Facility type, *n* (%)			
Public	305 (13.1)	38 (7.9)	**0**.**001**
Private	1,637 (70.3)	280 (58.0)	**<0**.**001**
Faith-based	386 (16.6)	165 (34.2)	**<0**.**001**
Rural, *n* (%)	630 (26.8)	178 (36.9)	**<0**.**001**
Country, *n* (%)			
Tanzania	550 (23.4)	256 (53.0)	**<0**.**001**
Kenya	1,006 (42.8)	169 (35.0)	**0**.**002**
Nigeria	472 (20.1)	43 (8.9)	**<0**.**001**
Ghana	247 (10.5)	15 (3.1)	**<0**.**001**
Other^d^	78 (3.3)	0 (0)	**<0**.**001**

a Among the excluded facilities, for comparison of facility levels, only 2,350 facilities were included in the comparison analysis because two facilities had unavailable facility level data and one was a pharmacy; for comparisons of facility type, only 2,328 facilities were included in the analysis because twenty-five facilities had unavailable facility type data.

b Wilcoxon Rank-Sum Test was used to compare patients visits, staff and quality scores at baseline; and two sample test of proportions was used to compare percentages.

c Quartiles. Range of quartiles showed for excluded and included facilities.

d The country category “Other” refers to the sum of facilities located in Namibia, Liberia and Uganda.

IQR, interquartile range.

Statistically significant *P* values are shown in bold.

### Statistical analysis

All analyses were performed using Stata version 16.1 (StataCorp). Facilities' characteristics between excluded and included facilities, and between baseline and follow-up of included facilities, were compared using nonparametric Wilcoxon Rank-Sum (Mann–Whitney) test, and two-sample proportion tests. Associations between the change in SafeCare score and the change in the number of patient visits and staff were assessed in bivariate and multivariate linear regression analyses, adjusting for the following (potential) confounders: quality score at baseline stratified by quality score categories (quartiles), number of patient visits/staff at baseline, facility level (dispensary, healthcare centre, or primary hospital), facility ownership (private, public, or faith-based), location (rural or urban), the number of days between the two SafeCare assessments (follow-up time) that were used to calculate the change in score, and country (Tanzania, Ghana, Kenya, Nigeria). As the models include unobserved contextual factors not captured by these variables, the explained variance (adjusted R^2^) is moderate, consistent with associational models of this type.

Only for the regressions estimating the change in number of patient visits, we excluded five outliers with values larger than 5,000 or smaller than −5,000 for this outcome, resulting in an analysis sample of 478 facilities. Regressions run on the full sample (*n* = 483) for the change in number of patients visits are shown in Supplementary File S1: Patient visits full sample.

Our two outcome variables of interest, the change in number of patient visits and staff, respectively, were not significantly skewed but had a kurtosis value larger than three (Supplementary File S2: Supplementary analyses). Hence, to confirm the results of our ordinary linear regressions we performed nonparametric Kernel regressions as a robustness check (Supplementary File S2: Supplementary analyses). Kernel regression is a technique for non-linear regression that models the mean of the outcome conditional on the covariates, but that makes no assumptions of the functional form of the relationship between the outcome and the covariates.

Finally, we classified the facilities in improvers and non-improvers, with improvers defined as ≥5 points improvement from baseline; and non-improvers defined as <5 points improvement from baseline to compare the baseline characteristics of these two groups at baseline. We performed heterogeneity analyses to calculate a QI cut off from baseline to follow-up for this classification: improvers. Cut offs of 3-, 7-, and 10-point improvement were also tested (Supplementary File S3: Improvers cut-off) showing the same associations than the 5-point cut off, but the latter was chosen because it showed the more significant differences between the two groups.

## Results

### Description of changes in patient visits, staff numbers and quality scores from baseline to follow-up

**Figure 2:**
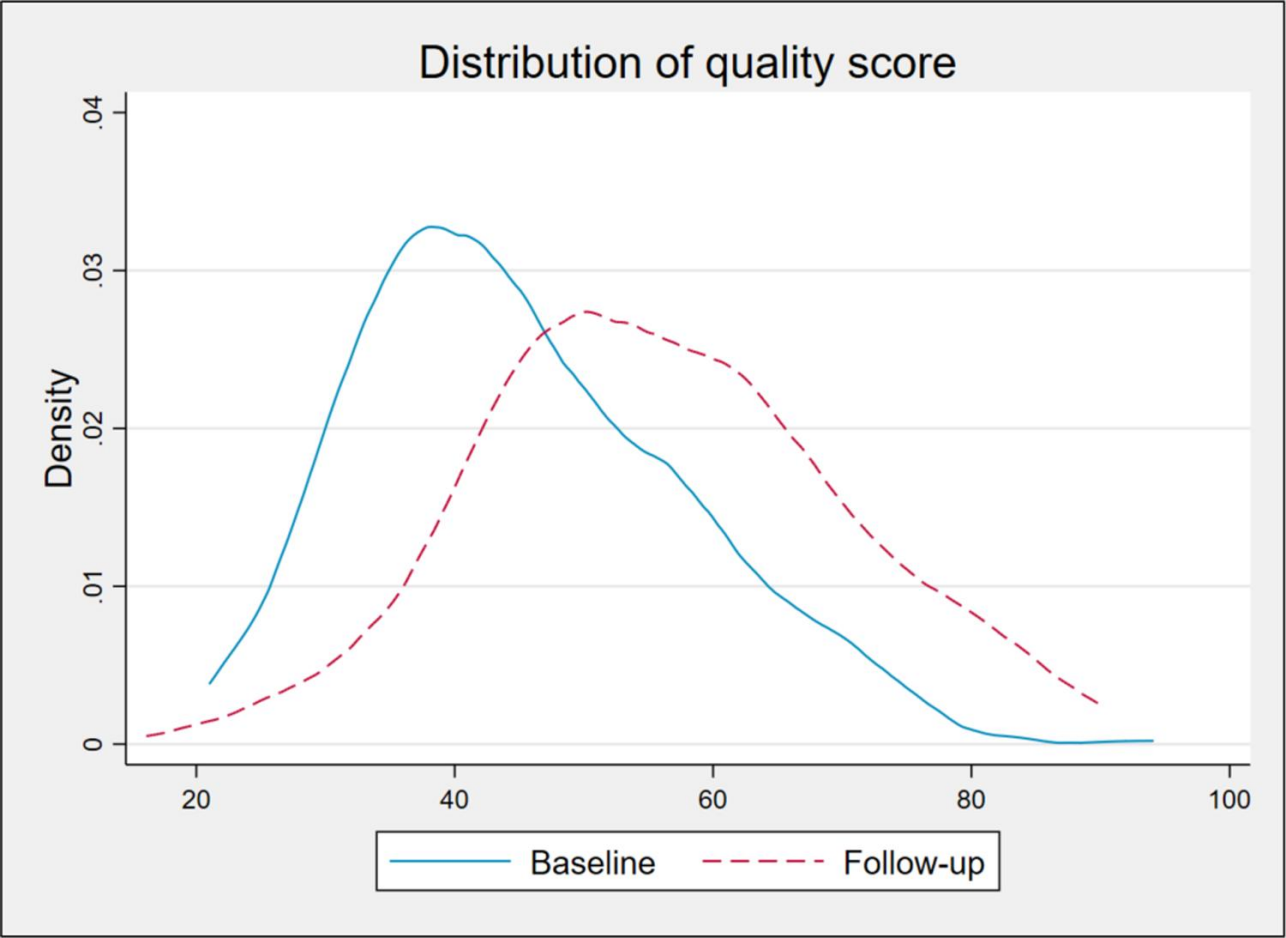
Distribution of quality score at baseline and follow-up. This figure shows that after ≥ 18 months follow-up, the proportion of facilities with a low and lower medium quality score (score <44) decreases, while the proportion of facilities with higher medium and high scores (score ≥ 44) increases, showing overall an improvement in quality in this group of facilities with time.

Table 2 compares the characteristics of the facilities in the analysis sample at baseline and follow-up. The median of the time to follow-up was 2.1 years, with an interquartile range of 1.1 years, and range of 1.5‒7.5 years. At baseline, facilities had a median of 600 patient visits per month and 15 employees. These overall numbers decreased to 557 patient visits per month and increase to 16 staff members at follow-up, but changes were not significant (*P* = 0.84/*P* = 0.44). In addition, the median quality score of 43.3 at baseline (at the cut-off between the lower-medium and higher-medium category by construction) improved significantly at follow-up to 55.4 (*P* < 0.001), with a significantly larger proportion of facilities falling in the high-quality category (from 24.8% to 52.8%, *P* < 0.001). Conversely, the proportion of facilities in the low and lower-medium quality categories decreased significantly at follow-up. Figure 2 shows the general improvement in the distribution of the quality scores.

**Table 2:** Facilities Characteristics at baseline and follow-up.

Variables	Baseline ^a^	Follow-up	*P-*value^a^
	(*N* = 483)	(*N* = 483)	
# Patient visits, median (IQR)	600 (1,000)	557 (1,230)	0.84
# Staff, median (IQR)	15 (28)	16 (31)	0.44
Quality score, median (IQR)	43.3 (18.3)	55.4 (19.1)	**<0**.**001**
Quality score categories, *n* (%)			
Low (21–36)	121 (25.1)	30 (6.2)	**<0**.**001**
Lower medium (36–43)	121 (24.1)	54 (11.2)	**<0**.**001**
Higher medium (43–54)	121 (25.1)	144 (29.8)	0.10
High (54–94)	120 (24.8)	255 (52.8)	**<0**.**001**

a Wilcoxon Rank-Sum Test was used to compare patients visits, staff and quality scores at baseline; and two sample test of proportions was used to compare percentages.

IQR, interquartile range.

Statistically significant *P* values are shown in bold.

### Association between the change in quality score and change in the number of patient visits

We found a significant positive association between the change in quality score and the change in patient visits from baseline to follow-up (Table 3 Column “Ordinary linear regression”). On average, a 1-point increase in quality score was associated with an increase of 7.9 patient visits per month (95% CI 1.7–14.1, *P* = 0.012). This association increased to 10.1 (95% CI 3.6–16.6, *P* = 0.002) after controlling for other predictor variables (Table 3 Column “Multiple linear regression”). Facilities that started in the medium-high or high-quality categories at baseline had significantly larger increases in the number of patient visits compared to facilities that started in the low-quality category (*P* = 0.049/*P* = 0.046). There was also a negative significant association between the baseline number of patient visits and the increase in patient visits (*P* < 0.001). Primary hospitals in comparison with dispensaries (*P* < 0.001), and Ghanan facilities in comparison with Tanzanian (*P* = 0.001), were associated with significantly higher, respectively lower, increases in patient numbers between baseline and follow-up. Additionally, the longer the follow-up time, the higher the observed increase in patient numbers (*P* < 0.001).

**Table 3:** Linear regression models to estimate the association between the change in quality score and the change in patient visits.

Independent variables (Dependent variable: *Δ* patient visits)	Ordinary linear regression (*N* = 478)		Multiple linear regression (*N* = 478)		Heterogeneity analysis (*N* = 478)	
	*β* (95% CI)	*P value*	*β* (95% CI)	*P value*	*β* (95% CI)	*P value*
*Δ* quality score	7.9 (1.7–14.1)	**0.012**	10.1 (3.6–16.6)	**0**.**002**	−12.6 (−30.4–5.2)	0.16
Baseline quality score						
Low (21–36)			Reference		Reference	
Lower medium (36–43)			67.2 (−151.4–285.8)	0.55	−91.6 (−444.0–260.7)	0.61
Higher medium (43–54)			236.5 (1.3–471.6)	**0**.**049**	40.5 (−300.4–381.3)	0.82
High (54–94)			267.0 (5.1–528.8)	**0**.**046**	76.6 (−266.3–419.5)	0.66
Baseline patient visits			−0.25 (−0.31 to −0.20)	**<0.001**	−0.24 (−0.31 to −0.17)	**<0.001**
Facility level						
Dispensary			Reference		Reference	
Healthcare center			75.0 (−102.4−252.3)	0.41	66.7 (−110.5−243.9)	0.46
Primary hospital			608.6 (348.9–868.2)	**<0.001**	587.9 (329.0–846.7)	**<0.001**
Facility type						
Private			Reference		Reference	
Public			−170.9 (−486.8–145.0)	0.29	−171.2 (−487.3–144.9)	0.29
Faith-based			72.0 (−140.6−284.7)	0.51	59.0 (−155.3−273.32)	0.59
Rural (reference: urban)			−149.6 (−337.8–38.6)	0.12	−152.0 (−339.9–35.9)	0.11
Country						
Tanzania			Reference		Reference	
Ghana			−786.2 (−1,249.8 to −322.5)	**0**.**001**	−812.5 (−1,274.4 to −350.7)	**0**.**001**
Kenya			121.7 (−81.1−324.5)	0.24	113.3 (−88.9−315.5)	0.27
Nigeria			−264.6 (−581.0−51.9)	0.10	−284.6 (−602.6−33.3)	0.08
Follow-up time (days)			0.4 (0.2−0.6)	**<0.001**	0.26 (0.03−0.49)	**0**.**025**
*Δ* quality score*lower medium baseline quality score					9.0 (−9.5−27.4)	0.34
*Δ* quality score*higher medium baseline quality score					13.5 (−3.9−30.8)	0.13
*Δ* quality score*high baseline quality score					16.6 (−1.4−34.6)	0.07
*Δ* quality score*baseline patient visits					−0.002 (−0.005−0.001)	0.21
*Δ* quality score*follow-up time					0.014 (0.003−0.025)	**0**.**015**
Adjusted R^2^	0.01		0.21		0.22	

*Δ*, change; *β*, regression coefficient that represents the average increase in the dependent variable (*Δ* patient visits) per each unit increase in the corresponding independent variable with all other variables held constant (after adjusting for all other variables in the model); CI, confidence interval; SD, standard deviation; Adjusted R^2^, indicates the proportion of the variance of the dependent variable (*Δ* patient visits) that can be explained by the independent variable after adjusting for all other variables in the model.

Statistically significant *P* values are shown in bold.

Heterogeneity analyses suggest that the positive association between the change in SafeCare score and change in the number of patient visits did not differ by baseline quality category. The association was stronger for longer follow-up times, i.e., when the baseline and follow-up surveys (20) were further apart (Table 3 Column “Heterogeneity analysis”). Finally, we replaced the change in SafeCare scores with the change in the alternative quality variable based on the subset of 38 business performance criteria. Findings are comparable (Supplementary File S4: Regressions business scores). An increase of 1 point in business performance score was associated with an increase of 6.3 patient visits (95% CI 1.0 ‒ 11.6, *P* = 0.019) in the bivariate regression and with an increase of 8.0 patient visits (95% CI 2.2–13.8, *P* = 0.007) in the multivariate regression.

### Association between the change in quality score and the change in staff numbers

Similar to the previous model, we also found a positive significant association between the improvement in quality scores and the increase in staff numbers (Table 4 Column “Ordinary linear regression”), that remained significant after controlling for other variables (Table 4 Column “Multiple linear regression”). A 1-point increase in quality score was associated with an average increase of 0.25 staff members (95% CI 0.13–1.38, *P* < 0.001). Facilities in the high-quality category at baseline experienced a larger increase in staff numbers compared to facilities in the low baseline category (*P* < 0.001). There was a negative association between the baseline number of staff and the increase in staff numbers (*P* < 0.001). Primary hospitals experienced significantly larger increases in staff numbers than dispensaries (*P* = 0.001). The change in staff numbers was not significantly associated with the ownership or the urban-rural location of the facility, nor to the country or the time to follow-up.

**Table 4:** Linear regression models to estimate the association between the change in quality score and the change in number of staff.

Independent variables (Dependent variable: *Δ* staff)	Ordinary linear regression (*N* = 483)		Multiple linear regression (*N* = 483)		Heterogeneity analysis (*N* = 483)	
	β (95% CI)	*P* value	β (95% CI)	*P* value	β (95% CI)	*P* value
*Δ* quality score	0.15 (0.03–0.26)	**0.011**	0.25 (0.13–0.38)	**<0.001**	−0.03(−0.4−0.3)	0.86
Baseline quality score						
Low (21–36)			Reference		Reference	
Lower medium (36–43)			1.2 (−3.0−5.4)	0.58	-0.8 (−7.7−6.0)	0.81
Higher medium (43−54)			4.0 (−0.6−8.5)	0.09	2.6 (−4.0−9.2)	0.44
High (54–94)			9.7 (4.6–14.7)	**<0.001**	7.8 (1.1–14.4)	**0**.**022**
Baseline staff			-0.1 (−0.2 to −0.1)	**<0.001**	-0.2 (−0.2 to −0.1)	**<0.001**
Facility level						
Dispensary			Reference		Reference	
Healthcare center			3.3 (−0.2−6.7)	0.07	3.5 (0.1–7.0)	**0**.**046**
Primary hospital			9.8 (3.8–15.8)	**0**.**001**	9.6 (3.7–15.6)	**0**.**002**
Facility type						
Private			Reference		Reference	
Public			−2.3 (−8.4−3.9)	0.47	−2.9 (−9.1−3.3)	0.36
Faith-based			1.5 (−2.7−5.7)	0.48	1.3 (−2.9−5.5)	0.54
Rural (reference: urban)			−0.4 (−4.0−3.2)	0.81	−0.3 (−3.9−3.3)	0.86
Country						
Tanzania			Reference		Reference	
Ghana			−0.4 (−9.3−8.5)	0.93	−0.4 (−9.2−8.5)	0.94
Kenya			2.6 (−1.3−6.5)	0.19	2.8 (−1.1−6.7)	0.16
Nigeria			−3.6 (−9.7−2.6)	0.25	−4.0 (−10.2−2.2)	0.21
Follow-up time (days)			0.002 (−0.002−0.006)	0.25	0.001 (−0.004−0.005)	0.7
*Δ* quality score*lower medium baseline quality score					0.1 (−0.3−0.5)	0.6
*Δ* quality score*higher medium baseline quality score					-0.0 (−0.4−0.3)	0.93
*Δ* quality score*high baseline quality score					0.2 (−0.2−0.5)	0.31
*Δ* quality score*baseline staff					0.003 (0.0003−0.005)	**0**.**026**
*Δ* quality score*follow-up time					0.0001 (−0.0001−0.0003)	0.26
Adjusted R^2^	0.01		0.1		0.12	

*Δ*, change; *β*, regression coefficient that represents the average increase in the dependent variable (*Δ* patient visits) per each unit increase in the corresponding independent variable with all other variables held constant (after adjusting for all other variables in the model); CI, confidence interval; SD, standard deviation; Adjusted R^2^, indicates the proportion of the variance of the dependent variable (*Δ* patient visits) that can be explained by the independent variable after adjusting for all other variables in the model.

Statistically significant *P* values are shown in bold.

The heterogeneity analysis showed that the positive association between a change in SafeCare scores and a change in staff numbers was stronger for larger facilities (with higher staff numbers at baseline) (*P* = 0.026) (Table 4 Column “Heterogeneity analysis”). Using the quality measure based on the 38 business performance criteria rather than the full SafeCare score yields similar results (Supplementary File S4: Regressions business score).

### Improvers vs. non-improvers

Table 5 compares the baseline characteristics of the facilities that achieved an improvement of 5 points or higher in the SafeCare quality score at follow-up [n [%], 344 [70.1]], with the facilities that did not achieve this level of improvement [147 (29.9)], classified as improvers and non-improvers, respectively. Improvers started from a significantly lower quality score at baseline compared to non-improvers [42.6 vs. (vs.) 51.1, *P* < 0.001] and they were significantly more likely to belong to the low-quality category (34.3% vs. 8.2%, *P* < 0.001). Regarding facility levels, the proportion of primary hospitals among the improvers was significantly higher at 21.5% vs. the non-improvers at 13.6% (*P* = 0.042). Improvers were also significantly more likely to be a public or faith-based facility. Finally, the proportion of Tanzanian and Nigerian facilities were significantly lower among the improvers.

**Table 5:** Baseline facility characteristics for improvers vs. non-improvers.

Variables	Improvers^a^	Non-improvers^b^	*P*-value^c^
	(*N* = 336)	(*N* = 147)	
# Patient visits, median (IQR)	601.5 (1,032.5)	500 (841)	**0**.**04**
# Staff, median (IQR)	16.8 (30)	13 (20)	**0**.**04**
Score at baseline, median (IQR)	41.1 (17.3)	48.7 (18.0)	**<0**.**001**
Quality score categories, *n* (%)			
Low (21–36)	111 (34.3)	10 (8.2)	**<0**.**001**
Lower medium (36–43)	86 (26.2)	35 (25.2)	0.676
Higher medium (43–54)	74 (22.4)	47 (31.3)	**0**.**02**
High (54–94)	65 (17.2)	55 (35.4)	**<0**.**001**
Facility level, *n* (%)			
Dispensary	142 (42.3)	65 (44.2)	0.689
Healthcare center	126 (37.5)	62 (42.2)	0.332
Primary hospital	68 (20.2)	20 (13.6)	**0**.**041**
Facility type, *n* (%)			
Public	26 (7.7)	12 (8.2)	0.875
Private	179 (53.3)	101 (68.7)	**0**.**002**
Faith-based	131 (39.0)	34 (23.1)	**<0**.**001**
Rural, *n* (%)	128 (38.1)	50 (34.0)	0.391
Country, *n* (%)			
Tanzania	195 (58.0)	61 (41.5)	**<0**.**001**
Ghana	12 (3.6)	3 (2.0)	0.372
Kenya	109 (32.4)	60 (40.8)	0.076
Nigeria	20 (6.0)	23 (15.7)	**<0**.**001**

a Improvers, facilities that had at the follow-up a *Δ* quality score ≥ 5.

b Non-improvers, facilities that had at the follow-up a *Δ* quality score <5.

c Wilcoxon Rank-Sum Test was used to compare patients visits, staff and quality scores at baseline; and two sample test of proportions was used to compare percentages.

*Δ*, change; SD, standard deviation.

Statistically significant *P* values are shown in bold.

## Discussion

UHC will produce better health outcomes in LMICs only on the foundations of a high-quality health system (1). In Africa, among the biggest impediments to improve quality of care in small- and medium- size public and private facilities is the limited investment in quality-of-care improvements and regulation from the healthcare authorities. As a result, millions of people die every year in LMICs due to the poor quality of care they receive (26). Evidence indicates that substandard care wastes significant economic and human resources due to for instance duplicated services, ineffective care, and avoidable hospital admissions (2). Hence, good quality health services not only ensure healthier societies but also healthier economies. It is a fallacy that quality of care is a luxury only rich countries can afford, since LMICs, especially the poorest ones, cannot afford the high cost of lack of quality care (2).

Before turning to our findings, it is important to clarify what associations of this kind can and cannot tell us. A facility's capacity to invest in QI is itself shaped by its financial position: facilities with more resources and stronger managerial capacity may be better placed to invest in quality, rather than quality investment being the driver of subsequent business performance. Our data do not allow us to rule out this alternative pathway. We note that, in the absence of an untreated comparison group (given that all facilities in our sample developed a QIP following their baseline assessment), approaches such as difference-in-differences or propensity score matching could not be applied, nor was an instrument available that plausibly affects QI without also affecting patient or staff numbers through other channels. We therefore interpret our findings as associations that are consistent with a business case for QI, rather than as evidence of a causal effect, and discuss them with this caveat throughout.

To enable the financial investment in their facilities, as well as create efficiency in care delivery, public and private (not-)for-profit facilities need to attract more clients to increase revenues to invest in QI. In turn, higher quality may lead to increased retention of patients, further enhancing revenues. But in a facility with limited resources the question arises whether these investments are worth it. Would small- and medium-sized facilities indeed realize an improvement in business performance when investing in QI?

Here, we quantify the (potential) business case for QI in SSA by analysing the QI journey of 483 facilities in four African countries. We correlate the changes in quality assessments as measured by SafeCare scores with changes in patients visits and staff numbers, used as proxies for business performance. We find that improvements in quality scores are significantly associated with increases in both patient visits (*β* = 10.1, 95% CI 3.6–16.6) and staff numbers (*β* = 0.25, 95% CI 0.13–0.38), suggesting a positive association between QI and business performance overall.

We further examined whether this association varies by facilities' baseline quality level. For patient visits, the estimated association appears to increase with baseline quality quartile, with a stronger effect of quality improvement among facilities that started with higher baseline quality; however, the interaction terms do not reach conventional levels of statistical significance, and this pattern should be considered suggestive rather than conclusive. No comparable pattern is observed for staff numbers. Further research, ideally with larger samples, is needed to establish whether the association between quality improvement and business performance indeed varies by baseline quality level.

As noted above, these associations do not imply a causal effect of QI on the number of patients and staff. Rather, two non-exclusive mechanisms could underlie this pattern. QI may increase trust and generate word-of-mouth recommendations among patients, which could subsequently translate into a higher demand for services and larger revenues. Alternatively, facilities with already better business performance and increasing revenues may simply have greater capacity to invest in the quality of the care they provide. Both pathways could reinforce each other over time, though our design cannot distinguish between them.

These findings pose a dilemma for financing QI within African healthcare systems that should be of concern for policy-makers and other stakeholders. In our cohort of 483 facilities, a quarter of facilities had a SafeCare score of 36 or less at baseline (low quality score category) (Table 1). When facilities deliver such low-quality care, decision-makers might be induced to close them, while at the same time these facilities may be the only provider for many in the surrounding communities.

In addition, many of the low scoring facilities in our sample worked hard to significantly improve their quality over time—as indicated by the large upward shift across quality categories from baseline to follow-up. A critical infrastructure and a basic level of human resources might be necessary conditions for QI to coincide with stronger business performance. This is also reflected in the higher proportion of primary hospitals among the improvers vs. the non-improvers. Therefore, substantial financial support and continued technical assistance may be needed for the facilities at the lower end of the quality distribution to break out of this low level of performance. In addition, better contracting, regulation, central supply management, and innovative models might enable them to continue their QI journey, to prevent harming the patients they attend, and to help them succeed as small/medium businesses and keep investing in quality. If higher quality of care is indeed associated with a higher willingness of patients to pay for the healthcare services they receive, this could in turn support facilities' capacity to invest.

The local private health sector is often underutilized in UHC strategies in SSA, despite its pivotal role in serving half of the population (27). However, during the COVID-19 pandemic in Africa, several successful public-private collaborations took place (28). As a result, local policy-makers are currently considering whether to make the inclusion of the private health sector the norm in health interventions. Public-private partnerships have several advantages that include joining complementary economic and human resources, but also making technology and know-how mutually available and creating more efficient referral systems. In order to achieve UHC and high quality of care, it seems essential to include the private sector in improvement and investment strategies.

To finance QI, digital loans may be an affordable and accessible financing alternative to the traditional loans from banks or government organizations since many facilities' owners do not have the collateral required to apply for bank loans (13).

Digital loans instead do not require assets and have a flexible and transparent re-payment method. Furthermore, during times of public health emergencies, such as the COVID-29 pandemic, which disrupt the functioning of the financial system, digital loans are able to provide continued financial support to healthcare facilities.

One size does not fit all. QI programs in LMICs should be customized to the needs of each geographical setting with a clear eye on availability of resources. When available, budgets are limited. Investments in QI could concentrate on those (public and private) healthcare providers with sufficient baseline capacity, given their potential to further realize both quality and business performance gains, and where senior staff demonstrate positive attitudes towards quality investments. Importantly, policy-makers should ensure adequate regulation and effective contracting to certify that key quality areas are targeted and sustainably addressed.

This study has several limitations. First, participating facilities were not randomly selected: they self-selected into the SafeCare program, and therefore may differ from non-participating facilities in their interest in QI. As shown in Table 1, facilities included in our analysis were, on average, larger, had slightly higher baseline quality scores, and were more likely to be dispensaries or primary hospitals, and of faith-based ownership, compared with the total study population. This suggests our sample may be skewed towards facilities with greater baseline managerial and resource capacity, which could inflate the associations reported here relative to a more representative sample, although it may also better capture the dynamics relevant to facilities already positioned to benefit from quality investment. The sample was also heavily concentrated in Tanzania (53%) and Kenya (35%), with limited representation from Nigeria (9%) and Ghana (3%), so the findings may primarily reflect dynamics in these two East-African countries rather than being representative of Sub-Saharan Africa as a whole. These characteristics should be taken into account when interpreting our findings. Second, as discussed above, our observational data do not allow us to establish a causal relationship between QI and business performance. Future research could consider an experimental design with random assignment of the QI program.

Third, as discussed in the Methods, although previous studies support our rationale for using number of patients and staff as business indicators, this approach has caveats: staff growth does not always indicate improved financial performance, and may instead reflect organizational inefficiencies or rising operating costs, while patient volume—though a stronger proxy—relies on the assumption that higher patient numbers do in fact reflect higher patient satisfaction in the facilities we study.

Finally, the implementation of quality plans involves a behavioural change. The coaching required to implement the QIPs requires sufficient human resources and economic investment (29–31). On the one hand, this may deny smallest facilities with least resources the possibility of implementing QIPs. On the other hand, this might imply that learnings from QI assessments are not automatically translated into QI (32). This is also reflected in the big gaps found in the literature between provider knowledge and practice (33, 34). The implementation of such processes can be importantly influenced by the intention to improve the quality of care that for some facilities is driven by altruistic motivation and a sense of duty, while for others such changes may be seen as sound financial investment.

## Conclusions

The significant positive associations found between QI and our business performance proxies—patients visits and staff numbers—suggest a potential business case for QI in SSA, although causality cannot be established. We hypothesize that this could translate into increasing revenues from growing patient numbers, allowing for further investments in the quality of care, and these two pathways may reinforce one another over time. Facilities that have started to implement QI may need financial support to accelerate their progress, particularly where baseline resources or capacity are limited. Public-private partnerships, and innovative loan schemes could help these facilities pursue QI and the business benefits potentially associated with it.

## Data Availability

The data analyzed in this study is subject to the following licenses/restrictions: the data are available upon request. Requests to access these datasets should be directed to w.janssens@vu.nl.
