# Supplementary material for: The business of quality: analysing the association between healthcare quality improvements and patient utilization and staff numbers in sub-Saharan Africa
Source: Front Health Serv. 2026 Aug 4;6:1856921. doi: 10.3389/frhs.2026.1856921 (PMC13481865; doi:10.3389/frhs.2026.1856921)
Supplement: Supplementary file 1 [file Table1.docx]

**The Business of Quality: Analysing the Association Between Healthcare Quality Improvements and Patient Utilization and Staff Numbers in Sub-Saharan Africa**

Gloria P. Gómez-Pérez^1,2^, Daniëlla Brals^3^, Aafke E. de Graaff^1^, Albena Sotirova^2^, Ibironke Dada^4^, Bonifacia Benefo Agyei^5^, Peter Risha^6^, Elizabeth Bonareri^7^, John T. Dekker^1^, Tobias F. Rinke de Wit^1,2,3^, Nicole Spieker^1^, Wendy Janssens^2,8*^

^1^ PharmAccess Foundation, Amsterdam, The Netherlands; ^2^ Amsterdam Institute for Global Health and Development, Amsterdam, The Netherlands; ^3^ Amsterdam University Medical Centers, location University of Amsterdam, Department of Global Health, Amsterdam, The Netherlands; ^4^ PharmAccess Nigeria, Lagos, Nigeria; ^5^ PharmAccess Ghana, Accra, Ghana; ^6^ PharmAccess Tanzania, Dar es Salaam, Tanzania; ^7^ PharmAccess Kenya, Nairobi, Kenya; ^8^ Faculty of Economics, Vrije Universiteit, Amsterdam, The Netherlands

*Corresponding author: Wendy Janssens, Amsterdam Institute for Global Health and Development, Amsterdam, The Netherlands. Email: w.janssens@vu.nl.

**Supplementary File 1: Patient visits full sample.**

*Abbreviations*: dNpatients, change in number of patients’ visits; dTotalNstaff, change in number of staff; dscore, change in SafeCare score; sd, standard deviation; p50, median; iqr, interquartile range; N, total number of observations.

This supplementary file contains descriptive statistics (Table S1, Figure S1, Figure S2) of the outcome variable change in patient visits with the original sample size of 483, i.e. before removing the outliers; and the corresponding regression models (Table S1). The five outliers had the following values for dNpatients and SafeCare quality scores at baseline (bSC):

1. dNpatients =16260, bSC = 89 (primary hospital).
2. dNpatients =5646, bSC = 35 (healthcare centre).
3. dNpatients =7187, bSC = 40 (primary hospital).
4. dNpatients = -9249, bSC = 68 (primary hospital).
5. dNpatients = -7453, bSC = 68 (primary hospital).

**Table S1**. Descriptive statistics change in number of patient visits n=483

**Figure S1**. Histogram of change in number patient visits (n=483) **Figure S2**. Boxplot of change in number patient visits (n=483)

**Table S2**. Linear regression models to estimate the association between the change in quality score and the change in patient visits with n=483

| **Independent variables**  (Dependent variable: Δ patient visits) | **Ordinary linear regression** (N = 483) | | **Multiple linear regression**  (N = 483) | | | **Heterogeneity analysis** (N = 483) | | | |
| --- | --- | --- | --- | --- | --- | --- | --- | --- | --- |
|  | β (95% CI) | *P value* | β (95% CI) | | *P value* | | β (95% CI) | *P value* | |
| Δ quality score | 14.6 (5.6−23.6) | **0.002** | 18.8 (9.1−28.4) | **< 0.001** | | | -10.4 (-37.0−16.2) | 0.44 | |
| Baseline quality score |  |  |  | |  | |  |  | |
| Low (21−36) |  |  | Reference | |  | | Reference |  | |
| Lower medium (36−43) |  |  | 143.2 (-182.8−469.2) | | 0.39 | | -168.3 (-695.4−358.9) | 0.53 | |
| Higher medium (43−54) |  |  | 305.7 (-45.2−656.5) | | 0.09 | | 26.9 (-482.9−536.7) | 0.92 | |
| High (54−94) |  |  | 454.4 (63.9−844.9) | | **0.023** | | 78.1 (-434.5−590.8) | 0.77 | |
| Baseline patient visits |  |  | -0.4 (-0.5 − -0.3) | | **< 0.001** | | -0.4 (-0.5 − -0.3) | **< 0.001** | |
| Facility level |  |  |  | |  | |  |  | |
| Dispensary |  |  | Reference | |  | | Reference |  | |
| Healthcare center |  |  | 102.6 (-162.6−367.9) | | 0.45 | | 120.6 (-144.2−385.4) | 0.37 | |
| Primary hospital |  |  | 899.0 (511.7−1286.3) | | **< 0.001** | | 881.2 (495.4−1267.0) | **< 0.001** | |
| Facility type |  |  |  | |  | |  |  | |
| Private |  |  | Reference | |  | | Reference |  | |
| Public |  |  | -82.6 (-555.4−390.3) | | 0.73 | | -93.2 (-565.8−379.5) | 0.70 | |
| Faith-based |  |  | 195.0 (-122.9−512.9) | | 0.23 | | 179.4 (-140.4−499.2) | 0.27 | |
| Rural (reference: urban) |  |  | -298.6 (-578.3 − -18.9) | | **0.036** | | -310.9 (-582.9 − -31.9) | **0.029** | |
| Country |  |  |  | |  | |  |  | |
| Tanzania |  |  | Reference | |  | | Reference |  | |
| Ghana |  |  | -570.8 (-1263.5−121.8) | | 0.11 | | -585.2 (-1274.5−104.2) | | 0.10 |
| Kenya |  |  | 307.0 (5.7−608.4) | | **0.046** | | 313.8 (13.7−613.9) | **0.04** | |
| Nigeria |  |  | -105.4 (-579.2−368.5) | | 0.66 | | -127.5 (-603.1−348.0) | 0.60 | |
| Follow-up time (days) |  |  | 0.3 (0.0−0.6) | | **0.037** | | 0.25 (-0.1−0.6) | 0.15 | |
| Δ quality score*lower medium baseline quality score |  |  |  | |  | | 16.8 (-10.8−44.4) | 0.23 | |
| Δ quality score*higher medium baseline quality score |  |  |  | |  | | 12.6 (-13.4−38.5) | 0.34 | |
| Δ quality score*high baseline quality score |  |  |  | |  | | 34.6 (7.9−61.4) | **0.011** | |
| Δ quality score*baseline patient visits |  |  |  | |  | | 0.001 (-0.003−0.005) | 0.58 | |
| Δ quality score*follow-up time |  |  |  | |  | | 0.01 (-0.01−0.03) | 0.22 | |
| Adjusted R^2^ | 0.02 | | 0.19 | | | | 0.20 | | |

*Abbreviations*: Δ, change; β, regression coefficient that represents the average increase in the dependent variable (Δ patient visits) per each unit increase in the corresponding independent variable with all other variables held constant (after adjusting for all other variables in the model); CI, confidence interval; SD, standard deviation; Adjusted R^2^, indicates the proportion of the variance of the dependent variable (Δ patient visits) that can be explained by the independent variable after adjusting for all other variables in the model.
