# Supplementary material for: The business of quality: analysing the association between healthcare quality improvements and patient utilization and staff numbers in sub-Saharan Africa
Source: Front Health Serv. 2026 Aug 4;6:1856921. doi: 10.3389/frhs.2026.1856921 (PMC13481865; doi:10.3389/frhs.2026.1856921)
Supplement: Supplementary file 2 [file Table2.docx]

**The Business of Quality: Analysing the Association Between Healthcare Quality Improvements and Patient Utilization and Staff Numbers in Sub-Saharan Africa**

Gloria P. Gómez-Pérez^1,2^, Daniëlla Brals^3^, Aafke E. de Graaff^1^, Albena Sotirova^2^, Ibironke Dada^4^, Bonifacia Benefo Agyei^5^, Peter Risha^6^, Elizabeth Bonareri^7^, John T. Dekker^1^, Tobias F. Rinke de Wit^1,2,3^, Nicole Spieker^1s^, Wendy Janssens^2,8*^

^1^ PharmAccess Foundation, Amsterdam, The Netherlands; ^2^ Amsterdam Institute for Global Health and Development, Amsterdam, The Netherlands; ^3^ Amsterdam University Medical Centers, location University of Amsterdam, Department of Global Health, Amsterdam, The Netherlands; ^4^ PharmAccess Nigeria, Lagos, Nigeria; ^5^ PharmAccess Ghana, Accra, Ghana; ^6^ PharmAccess Tanzania, Dar es Salaam, Tanzania; ^7^ PharmAccess Kenya, Nairobi, Kenya; ^8^ Faculty of Economics, Vrije Universiteit, Amsterdam, The Netherlands

*Corresponding author: Wendy Janssens, Amsterdam Institute for Global Health and Development, Amsterdam, The Netherlands. Email: w.janssens@vu.nl.

**Supplementary file 2: Supplementary Analyses**

*Abbreviations*: dNpatients, change in number of patients’ visits; dTotalNstaff, change in number of staff; dscore, change in SafeCare score; sd, standard deviation; p50, median; iqr, interquartile range; N, total number of observations.

This file contains descriptive statistics of outcome variables (Table S3, Table S4, Figure S3, Figure S4, Figure S5, Figure S6) and nonparametric Kernel regression robustness check (Table S5, Table S6, Figure S7, Figure S8).

1. **Descriptive statistics of outcome variables**

**Table S3**. Description of change in patient visits data

**Figure S3**. Histogram of change in number of patient visits **Figure S4**. Boxplot of change in number of patient visits

**Table S4**. Description of change in the number of staff data

**Figure S3**. Histogram of the change in the number of staff **Figure S4**. Boxplot of the change in the number of staff

**Figure S5**. Two-way scatter graph of the change in the number of patients and change in SafeCare score.

**Figure S6**. Two-way scatter graph of the change in the number of staff and change in SafeCare score.

2. **Robustness check of linear regressions with nonparametric Kernel regressions**

Kernel regression is a technique for non-linear regressions. Like linear regression, nonparametric regression models the mean of the outcome conditional on the covariates, but unlike linear regression makes no assumptions of the functional form of the relationship between the outcome and the covariates.^[1]^

**Table S5**. Nonparametric Kernel regression of the change in number of patients and change in SafeCare score.

477 out 478 observations were taken by the Kernel regression model. The coefficient table reports the average of the predicted means and the average of the predicted derivatives of the mean function with bootstrap standard errors. The average of the observation-level predicted (dNpatients) is 88.14. The average of the observation-level marginal effects is 7.04, which indicates that increasing dscore increases the mean number of dNpatients.

**Figure S7**. Graph of nonparametric Kernel regression of the change in number of patient visits and change in SafeCare score.

**Table S6**. Nonparametric Kernel regression of the change in number of staff and change in SafeCare score.

482 out 483 observations were taken by the Kernel regression model. The coefficient table reports the average of the predicted means and the average of the predicted derivatives of the mean function with bootstrap standard errors. The average of the observation-level predicted (dTotalNstaff) is 1.76. The average of the observation-level marginal effects is 0.15, which indicates that increasing dscore increases the mean number of dTotalNstaff.

**Figure S8**. Graph of nonparametric Kernel regression of the change in number of staff and change in SafeCare score.

1. Harvard Business School – Introduction to Kernel regressions, 2009 ([kernel.pdf (hbs.edu)](https://www.hbs.edu/research-computing-services/Shared%20Documents/Training/kernel.pdf); STATA Nonparametric Kernel regression (chrome-extension://efaidnbmnnnibpcajpcglclefindmkaj/https://www.stata.com/manuals/rnpregresskernel.pdf)
