# Supplementary material for: The business of quality: analysing the association between healthcare quality improvements and patient utilization and staff numbers in sub-Saharan Africa
Source: Front Health Serv. 2026 Aug 4;6:1856921. doi: 10.3389/frhs.2026.1856921 (PMC13481865; doi:10.3389/frhs.2026.1856921)
Supplement: Supplementary file 3 [file Table3.docx]

**The Business of Quality: Analysing the Association Between Healthcare Quality Improvements and Patient Utilization and Staff Numbers in Sub-Saharan Africa**

Gloria P. Gómez-Pérez^1,2^, Daniëlla Brals^3^, Aafke E. de Graaff^1^, Albena Sotirova^2^, Ibironke Dada^4^, Bonifacia Benefo Agyei^5^, Peter Risha^6^, Elizabeth Bonareri^7^, John T. Dekker^1^, Tobias F. Rinke de Wit^1,2,3^, Nicole Spieker^1^, Wendy Janssens^2,8*^

^1^ PharmAccess Foundation, Amsterdam, The Netherlands; ^2^ Amsterdam Institute for Global Health and Development, Amsterdam, The Netherlands; ^3^ Amsterdam University Medical Centers, location University of Amsterdam, Department of Global Health, Amsterdam, The Netherlands; ^4^ PharmAccess Nigeria, Lagos, Nigeria; ^5^ PharmAccess Ghana, Accra, Ghana; ^6^ PharmAccess Tanzania, Dar es Salaam, Tanzania; ^7^ PharmAccess Kenya, Nairobi, Kenya; ^8^ Faculty of Economics, Vrije Universiteit, Amsterdam, The Netherlands

*Corresponding author: Wendy Janssens, Amsterdam Institute for Global Health and Development, Amsterdam, The Netherlands. Email: w.janssens@vu.nl.

**Supplementary File 3: Improvers cut-off.**

This file describes the characteristics of the facilities at baseline comparing the different cut-offs studied for the definition of the improvers versus non-improvers: 3-, 5-, 7-, and 10-point improvement in SafeCare scores. The 5-point improvement cut-off was chosen for the final analysis.

**Table S8.** Baseline facility characteristics for improvers (Δ quality score ≥ 3) versus non-improvers (Δ quality score < 3).

| **Cut off at 3** |  |  |  |
| --- | --- | --- | --- |
|  | Improver | Non-improver | *P*-value |
|  | (N=367) | (N=124) |  |
| # Patient visits, mean (SD) | 1151.22 (1631.21) | 1023.23 (1714.8) | 0.456 |
| # Staff, mean (SD) | 35.54 (48.5) | 25.48 (41.24) | **0.039** |
| BasicScore | 42.89 (11.59) | 51.65 (12.43) | **<0.001** |
| *Quality score categories, n (%)* |  |  |  |
| Low | 112 (32.7) | 12 (8.1) | **<0.001** |
| Lower medium | 93 (27) | 33 (22.6) | 0.335 |
| Higher medium | 78 (22.6) | 47 (32.3) | **0.032** |
| High | 61 (17.7) | 55 (37.1) | **<0.001** |
| *Facility level, n (%)* |  |  |  |
| Dispensary | 143 (41.7) | 66 (45.2) | 0.5 |
| Health center | 127 (37.1) | 62 (41.9) | 0.335 |
| Primary hospital | 73 (21.3) | 19 (12.9) | **0.041** |
| *Facility type, n (%)* |  |  |  |
| Private | 25 (7.4) | 13 (8.9) | 0.586 |
| Public | 187 (54.5) | 102 (69.4) | **0.004** |
| Faith-based | 131 (38.1) | 32 (21.8) | **0.001** |
| Rural, n (%) | 128 (37.3) | 49 (33.1) | 0.394 |
| *Country, n (%)* |  |  |  |
| Tanzania | 194 (56.4) | 58 (39.5) | **0.001** |
| Ghana | 11 (3.3) | 4 (2.4) | 0.635 |
| Kenya | 110 (31.9) | 62 (41.9) | **0.042** |
| Nigeria | 22 (6.3) | 24 (16.1) | **0.001** |
| Other | 7 (2.2) | 0 (0) | 0.098 |

*Abbreviations*: Δ, change in SafeCare quality score from baseline to follow-up; SD, standard deviation.

**Table S9.** Baseline facility characteristics for improvers (Δ quality score ≥ 5) versus non-improvers (Δ quality score < 5).

| **Cut off at 5** |  |  |  |
| --- | --- | --- | --- |
|  | Improver | Non-improver | *P*-value |
|  | (N=344) | (N=147) |  |
| # Patient visits, mean (SD) | 1163.78 (1669.56) | 1013.84 (1610.48) | 0.358 |
| # Staff, mean (SD) | 35.56 (48.26) | 27.01 (43.24) | 0.065 |
| BasicScore | 42.55 (11.47) | 51.07 (12.48) | **<0.001** |
| *Quality score categories, n (%)* |  |  |  |
| Low | 118 (34.3) | 12 (8.2) | **<0.001** |
| Lower medium | 90 (26.2) | 37 (25.2) | 0.819 |
| Higher medium | 77 (22.4) | 46 (31.3) | **0.037** |
| High | 59 (17.2) | 52 (35.4) | **<0.001** |
| *Facility level, n (%)* |  |  |  |
| Dispensary | 144 (41.9) | 65 (44.2) | 0.629 |
| Health center | 126 (36.6) | 62 (42.2) | 0.248 |
| Primary hospital | 74 (21.5) | 20 (13.6) | **0.042** |
| *Facility type, n (%)* |  |  |  |
| Private | 26 (7.6) | 12 (8.2) | 0.819 |
| Public | 185 (53.8) | 101 (68.7) | **0.002** |
| Faith-based | 133 (38.7) | 34 (23.1) | **0.001** |
| Rural, n (%) | 128 (37.2) | 50 (34) | 0.501 |
| *Country, n (%)* |  |  |  |
| Tanzania | 195 (56.7) | 61 (41.5) | **0.002** |
| Ghana | 12 (3.5) | 3 (2) | 0.394 |
| Kenya | 109 (31.7) | 60 (40.8) | 0.051 |
| Nigeria | 20 (5.8) | 23 (15.6) | **<0.001** |
| Other | 8 (2.3) | 0 (0) | 0.063 |

*Abbreviations*: Δ, change in SafeCare quality score from baseline to follow-up; SD, standard deviation.

**Table S10.** Baseline facility characteristics for improvers (Δ quality score ≥ 7) versus non-improvers (Δ quality score < 7).

| **Cut off at 7** |  |  |  |
| --- | --- | --- | --- |
|  | Improver | Non-improver | P-value |
|  | (N=319) | (N=172) |  |
| # Patient visits, mean (SD) | 1194.14 (1722.75) | 979.34 (1506.58) | 0.17 |
| # Staff, mean (SD) | 35.79 (49.05) | 27.82 (42.39) | 0.072 |
| BasicScore | 42.14 (11.36) | 50.61 (12.39) | **<0.001** |
| *Quality score categories, n (%)* |  |  |  |
| Low | 122 (35.4) | 15 (9.9) | **<0.001** |
| Lower medium | 91 (26.3) | 37 (25) | 0.748 |
| Higher medium | 75 (21.9) | 45 (30.8) | **0.031** |
| High | 56 (16.3) | 50 (34.3) | **<0.001** |
| *Facility level, n (%)* |  |  |  |
| Dispensary | 137 (39.8) | 70 (47.7) | 0.093 |
| Health center | 130 (37.9) | 57 (39) | 0.825 |
| Primary hospital | 77 (22.3) | 20 (13.4) | **0.017** |
| *Facility type, n (%)* |  |  |  |
| Private | 26 (7.5) | 12 (8.1) | 0.808 |
| Public | 189 (54.9) | 95 (64.5) | **0.038** |
| Faith-based | 129 (37.6) | 40 (27.3) | **0.022** |
| Rural, n (%) | 127 (37) | 51 (34.9) | 0.644 |
| *Country, n (%)* |  |  |  |
| Tanzania | 194 (56.4) | 65 (44.2) | **0.009** |
| Ghana | 13 (3.8) | 3 (1.7) | 0.216 |
| Kenya | 111 (32.3) | 56 (38.4) | 0.177 |
| Nigeria | 18 (5.3) | 22 (15.1) | **<0.001** |
| Other | 8 (2.2) | 1 (0.6) | 0.179 |

*Abbreviations*: Δ, change in SafeCare quality score from baseline to follow-up; SD, standard deviation.

**Table S11.** Baseline facility characteristics for improvers (Δ quality score ≥ 10) versus non-improvers (Δ quality score < 10).

| **Cut off at 10** |  |  |  |
| --- | --- | --- | --- |
|  | Improver | Non-improver | *P*-value |
|  | (N=268) | (N=233) |  |
| # Patient visits, mean (SD) | 1224.74 (1782.01) | 991.7 (1474.39) | 0.12 |
| # Staff, mean (SD) | 36.49 (51) | 28.8 (41.25) | 0.07 |
| BasicScore | 41.53 (11.17) | 49.4 (12.46) | **<0.001** |
| *Quality score categories, n (%)* |  |  |  |
| Low | 128 (37.3) | 20 (13.5) | **<0.001** |
| Lower medium | 90 (26.1) | 38 (25.6) | 0.888 |
| Higher medium | 78 (22.8) | 41 (27.8) | 0.2 |
| High | 47 (13.8) | 49 (33.2) | **<0.001** |
| *Facility level, n (%)* |  |  |  |
| Dispensary | 139 (40.3) | 67 (45.3) | 0.266 |
| Health center | 127 (36.9) | 59 (39.9) | 0.501 |
| Primary hospital | 78 (22.8) | 22 (14.8) | **0.026** |
| *Facility type, n (%)* |  |  |  |
| Private | 27 (7.8) | 11 (7.6) | 0.93 |
| Public | 193 (56) | 90 (61) | 0.263 |
| Faith-based | 125 (36.2) | 46 (31.4) | 0.264 |
| Rural, n (%) | 118 (34.3) | 57 (38.6) | 0.332 |
| *Country, n (%)* |  |  |  |
| Tanzania | 193 (56) | 70 (47.5) | 0.063 |
| Ghana | 12 (3.4) | 4 (2.7) | 0.669 |
| Kenya | 112 (32.5) | 54 (36.8) | 0.318 |
| Nigeria | 19 (5.6) | 18 (12.6) | **0.007** |
| Other | 9 (2.6) | 1 (0.4) | **0.06** |

*Abbreviations*: Δ, change in SafeCare quality score from baseline to follow-up; SD, standard deviation.
