# Supplementary material for: The business of quality: analysing the association between healthcare quality improvements and patient utilization and staff numbers in sub-Saharan Africa
Source: Front Health Serv. 2026 Aug 4;6:1856921. doi: 10.3389/frhs.2026.1856921 (PMC13481865; doi:10.3389/frhs.2026.1856921)
Supplement: Supplementary file 4 [file Table4.docx]

**The Business of Quality: Analysing the Association Between Healthcare Quality Improvements and Patient Utilization and Staff Numbers in Sub-Saharan Africa**

Gloria P. Gómez-Pérez^1,2^, Daniëlla Brals^3^, Aafke E. de Graaff^1^, Albena Sotirova^2^, Ibironke Dada^4^, Bonifacia Benefo Agyei^5^, Peter Risha^6^, Elizabeth Bonareri^7^, John T. Dekker^1^, Tobias F. Rinke de Wit^1,2,3^, Nicole Spieker^1^, Wendy Janssens^2,8*^

^1^ PharmAccess Foundation, Amsterdam, The Netherlands; ^2^ Amsterdam Institute for Global Health and Development, Amsterdam, The Netherlands; ^3^ Amsterdam University Medical Centers, location University of Amsterdam, Department of Global Health, Amsterdam, The Netherlands; ^4^ PharmAccess Nigeria, Lagos, Nigeria; ^5^ PharmAccess Ghana, Accra, Ghana; ^6^ PharmAccess Tanzania, Dar es Salaam, Tanzania; ^7^ PharmAccess Kenya, Nairobi, Kenya; ^8^ Faculty of Economics, Vrije Universiteit, Amsterdam, The Netherlands

*Corresponding author: Wendy Janssens, Amsterdam Institute for Global Health and Development, Amsterdam, The Netherlands. Email: w.janssens@vu.nl.

**Supplementary File 4: Regressions business score.**

**Table S12**. Linear regression models to estimate the effect of the change in business performance quality scores on the change in patient visits.

| **Independent variables**  (Dependent variable: Δ patient visits) | **Ordinary linear regression**  (N = 478) | | **Multiple linear regression**   (N = 478) | | | **Heterogeneity analysis** (N = 478) | | | |
| --- | --- | --- | --- | --- | --- | --- | --- | --- | --- |
|  | β (95% CI) | *P value* | β (95% CI) | | *P value* | | β (95% CI) | *P value* | |
| Δ business performance score^1^ | 6.3 (1.0−11.6) | **0.019** | 8.0 (2.2−13.8) | **0.007** | | | 4.0 (-2.7−10.7) | 0.25 | |
| Baseline business performance score |  |  |  | |  | |  |  | |
| Low (1.0−5.0) |  |  | Reference | |  | | Reference |  | |
| Lower medium (5.5−11.5) |  |  | 93.3 (-142.5−329.2) | | 0.44 | | 88.3 (-193.1−369.6) | 0.54 | |
| Higher medium (12.0−27.8) |  |  | 155.7 (-84.5−396.0) | | 0.20 | | -42.3 (-350.7−266.0) | 0.78 | |
| High (28.0−73.0) |  |  | 249.3 (-37.3−535.9) | | 0.09 | | 297.3 (-58.1−652.8) | 0.10 | |
| Baseline patient visits |  |  | -0.25 (-0.3 − -0.19) | | **< 0.001** | | -0.25 (-0.31 − -0.18) | **< 0.001** | |
| Facility level |  |  |  | |  | |  |  | |
| Dispensary |  |  | Reference | |  | | Reference |  | |
| Healthcare center |  |  | 65.8 (-117.2−248.9) | | 0.48 | | 33.8 (-148.1−215.8) | 0.71 | |
| Primary hospital |  |  | 609.9 (329.4−890.4) | | **< 0.001** | | 585.9 (307.9−863.8) | **< 0.001** | |
| Facility type |  |  |  | |  | |  |  | |
| Private |  |  | Reference | |  | | Reference |  | |
| Public |  |  | -168.2 (-492.0−155.7) | | 0.31 | | -198.2 (-524.1−127.7) | 0.23 | |
| Faith-based |  |  | 68.3 (-145.8−282.4) | | 0.53 | | 91.0 (-122.0−303.9) | 0.40 | |
| Rural (reference: urban) |  |  | -137.9 (-328.7 – 53.0) | | 0.16 | | -154.4 (-343.7 – 34.9) | 0.11 | |
| Country |  |  |  | |  | |  |  | |
| Tanzania |  |  | Reference | |  | | Reference |  | |
| Ghana |  |  | -739.8 (-1213.3− -266.3) | | **0.002** | | -721.3 (-1190.6− -251.9) | | **0.003** |
| Kenya |  |  | 108.1 (-100.4−316.5) | | 0.31 | | 131.5 (-75.4−338.5) | 0.21 | |
| Nigeria |  |  | -208.4 (-542.0−125.2) | | 0.22 | | -179.5 (-510.2−151.1) | 0.29 | |
| Follow-up time (days) |  |  | 0.35 (0.14−0.57) | | **0.001** | | 0.32 (0.09−0.54) | **0.007** | |
| Δ quality score*lower medium baseline quality score |  |  |  | |  | | -3.1 (-17.0−10.9) | 0.66 | |
| Δ business performance score*higher medium baseline quality score |  |  |  | |  | | 18.5 (2.3−34.7) | **0.025** | |
| Δ business performance score*high baseline quality score |  |  |  | |  | | -8.3 (-26.4−9.8) | 0.37 | |
| Δ business performance score*baseline patient visits |  |  |  | |  | | -0.0003 (-0.003−0.003) | 0.84 | |
| Δ business performance score*follow-up time |  |  |  | |  | | 0.003 (-0.006− 0.012) | 0.51 | |
| Adjusted R^2^ | 0.01 | | 0.21 | | | | 0.23 | | |

^1^ For the models described in this table, only the quality scores of the criteria related to business performance were included in this analysis, which comprises 38 out of a total of 753 SafeCare criteria.

*Abbreviations*: Adjusted R^2^, indicates the proportion of the variance of the dependent variable (Δ patient visits) that can be explained by the independent variable after adjusting for all other variables in the model; Δ, change; β, regression coefficient that represents the average increase in the dependent variable (Δ patient visits) per each unit increase in the corresponding independent variable with all other variables held constant (after adjusting for all other variables in the model); CI, confidence interval; SD, standard deviation.

**Table S13**. Linear regression models to estimate the effect of the change in business performance quality scores on the change in number of staff.

| **Independent variables**  (Dependent variable: Δ staff) | **Ordinary linear regression**  (N = 483) | | | **Multiple linear regression**  (N = 483) | | | **Heterogeneity analysis** (N = 483) | |
| --- | --- | --- | --- | --- | --- | --- | --- | --- |
|  | β (95% CI) | *P* value | | β (95% CI) | | *P* value | β (95% CI) | *P* value |
| Δ business performance score^1^ | 0.16 (0.06−0.25) | | **0.001** | 0.2 (0.1−0.3) | **0.001** | | 0.13 (-0.01−0.25) | 0.06 |
| Baseline business performance score |  |  | |  | |  |  |  |
| Low (1.0−5.0) |  |  | | Reference | |  | Reference |  |
| Lower medium (5.5−11.5) |  |  | | 0.8 (-3.8−5.4) | | 0.73 | 1.6 (-3.9−7.2) | 0.56 |
| Higher medium (12.0−27.8) |  |  | | 1.2 (-3.5−5.9) | | 0.62 | 1.0 (-5.0−7.1) | 0.74 |
| High (28.0−73.0) |  |  | | 6.5 (0.9−12.1) | | **0.024** | 8.9 (1.9−16.0) | **0.013** |
| Baseline staff |  |  | | -0.12 (-0.16 − -0.7) | | **< 0.001** | -0.16 (-0.2 − -0.1) | **< 0.001** |
| Facility level |  |  | |  | |  |  |  |
| Dispensary |  |  | | Reference | |  | Reference |  |
| Healthcare center |  |  | | 2.8 (-0.8−6.4) | | 0.13 | 2.6 (-1.0−6.2) | 0.16 |
| Primary hospital |  |  | | 8.6 (2.4−14.9) | | **0.007** | 7.9 (1.7−14.2) | **0.013** |
| Facility type |  |  | |  | |  |  |  |
| Private |  |  | | Reference | |  | Reference |  |
| Public |  |  | | -2.9 (-9.2−3.4) | | 0.37 | -3.6 (-10.0−2.9) | 0.28 |
| Faith-based |  |  | | 1.2 (-3.0−5.4) | | 0.58 | 0.8 (-3.4−5.0) | 0.71 |
| Rural (reference: urban) |  |  | | -0.3 (-4.0−3.3) | | 0.87 | -0.1 (-3.8−3.6) | 0.96 |
| Country |  |  | |  | |  |  |  |
| Tanzania |  |  | | Reference | |  | Reference |  |
| Ghana |  |  | | 1.1 (-8.0−10.2) | | 0.81 | 1.3 (-7.8−10.4) | 0.78 |
| Kenya |  |  | | 2.4 (-1.7−6.4) | | 0.25 | 2.8 (-1.2−6.9) | 0.17 |
| Nigeria |  |  | | -2.0 (-8.5−4.5) | | 0.55 | -1.6 (-8.1−4.9) | 0.63 |
| Follow-up time (days) |  |  | | 0.003 (-0.001−0.01) | | 0.15 | 0.002 (-0.003−0.006) | 0.40 |
| Δ quality score*lower medium baseline quality score |  |  | |  | |  | -0.1 (-0.4−0.2) | 0.48 |
| Δ business performance score*higher medium baseline quality score |  |  | |  | |  | -0.03 (-0.3−0.3) | 0.87 |
| Δ business performance score*high baseline quality score |  |  | |  | |  | -0.2 (-0.6−0.1) | 0.20 |
| Δ business performance score*baseline staff |  |  | |  | |  | 0.004 (0.001−0.006) | **0.008** |
| Δ business performance score*follow-up time |  |  | |  | |  | 0.0001 (-0.0001−0.0002) | 0.48 |
| Adjusted R^2^ | 0.02 | | | 0.08 | | | 0.09 | |

^1^ For the models described in this table, only the quality scores of the criteria related to business performance were included in this analysis, which comprises 38 out of a total of 753 SafeCare criteria.

*Abbreviations*: Adjusted R2, indicates the proportion of the variance of the dependent variable (Δ patient visits) that can be explained by the independent variable after adjusting for all other variables in the model; Δ, change; β, regression coefficient that represents the average increase in the dependent variable (Δ patient visits) per each unit increase in the corresponding independent variable with all other variables held constant (after adjusting for all other variables in the model); CI, confidence interval; SD, standard deviation.
